# NACC1 accelerates the progression of AML by regulating the ADAM9/PI3K/AKT axis

**DOI:** 10.7150/ijms.102266

**Published:** 2025-01-06

**Authors:** Ying Zhang, Liang Zhong, Peng Wan, Yi Zhao, Meng Wang, Hongyan Zhang, Yang Liao, Ying Deng, Beizhong Liu

**Affiliations:** 1 Central Laboratory of Yongchuan Hospital, Chongqing Medical University, Chongqing 402160, China.; 2 Key Laboratory of Laboratory Medical Diagnostics, Ministry of Education, Department of Laboratory Medicine, Chongqing Medical University, Chongqing 400016, China.; 3 Clinical Laboratory of The Affiliated Rehabilitation Hospital, Chongqing Medical University, Chongqing 400050, China.

**Keywords:** Acute myeloid leukemia, NACC1, ADAM9, PI3K/AKT pathway, Proliferation, Cell apoptosis

## Abstract

Nucleus accumbens-associated protein 1 (NACC1) regulates various types of biological processes. It is a transcription factor associated with cancer. NACC1 is overexpressed in many human malignancies and can regulate the progression, metastasis, and drug resistance of cancer cells. However, its precise role in acute myeloid leukemia (AML) remains unknown. This study aimed to unravel the basic mechanism of NACC1 in AML. Our findings demonstrated that NACC1 is immensely expressed in AML cells. Lentiviral vector-mediated knockdown of NACC1 inhibited the PI3K/AKT signaling pathway. Simultaneously, NACC1 knockdown promoted apoptosis, suppressed the proliferative capacity of AML cells, and resulted in cell cycle arrest during the G0/G1 phase. Additionally, A disintegrin and metalloproteinase 9 (ADAM9) was markedly expressed in AML cells. NACC1 regulated ADAM9 expression. ADAM9 expression was also downregulated after NACC1 knockdown. Concurrently, ADAM9 knockdown affected the activity of AML cells by decelerating the growth rate, promoting apoptosis, and blocking cell cycle progression. In addition, the AKT activator SC79 restored the inhibited cell proliferation after NACC1 knockdown and ADAM9 knockdown. In conclusion, our study suggested that the NACC1/ADAM9/PI3K/AKT axis is crucial for sustaining the survival of AML cells, indicating that NACC1 may be a viable target for treating AML.

## Introduction

Acute myeloid leukemia (AML) affects myeloid hematopoietic stem cells. The clonal proliferation of abnormally differentiated mother cells from the bone marrow line is a hallmark of AML 1, 2. AML is a prominent type of leukemia in adult patients, and the prevalence gradually increases with age 3. The majority of patients do not experience substantial remission after initial treatment and develop refractory AML 4. The mortality rate of the disease is higher among elderly patients 5, 6. AML therapy has significantly advanced over the past years; however, patients with AML are at a higher risk of relapsing and are prone to medication resistance 7. Therefore, to explore new treatments for AML, more studies are needed on the molecular target of AML.

BTB is also frequently referred to as the POZ domain. It regulates many cellular functions and is a major motif involved in protein-protein interactions 8-11. BTB/POZ family proteins are involved in biological processes. They play a key role in transcriptional regulation 12. Previous studies have demonstrated its significance in the growth of many cancers and diseases 13-15. Multiple proteins containing this domain have been linked to human cancer, including B-cell lymphoma 6 (BCL-6) 16, promyelocytic leukemia zinc finger (PLZF) 17, 18, and zinc finger and BTB domain protein 1 (ZBTB1) 19.

Nucleus accumbens-associated protein 1 (NACC1), containing a BTB/POZ domain, is a transcription factor associated with cancer 20. Initially, NACC1 was discovered in the nucleus accumbens of cocaine-treated rats. The nucleus accumbens is a key region of the brain that is strongly linked to addictive behaviors 21. Moreover, NACC1 is closely linked to the pluripotency of embryonic stem cells, mediating various biological functions 22-25. Meanwhile, abnormal expression of NACC1 is linked to the treatment resistance of cancer cells 26, 27. Abnormal expression of NACC1 was shown to be linked to the poor prognostic features of many types of malignant tumors, such as ovarian cancer 28, 29, colorectal cancer 30, melanoma 31, 32, hepatocellular carcinoma 33 and other diseases. However, the expression level of NACC1 and its biological roles and pathogenic mechanism in AML is still unknown. Thus, it is needed to investigate NACC1 as a target for treating AML.

A disintegrin and metalloproteinases (ADAMs), a multi-domain family of proteins, are regarded as a prospective target for treating cancer 34. This protein family possesses multiple biological functions 35. As a member of the ADAMs family, ADAM9 is deemed a potential target for treating cancer 36. Many cancers, including ovarian cancer 37, lung cancer 38, 39, colorectal cancer 40, and stomach cancer 41, have ADAM9 overexpression. ADAM9 promotes cancer cells to migrate and grow, thereby contributing to tumor development and progression 42, 43. Our study proved that NACC1 regulates the expression of ADAM9. As a downstream effector of NACC1, ADAM9 is a potential target for AML treatment.

This research aimed to delve into the role of NACC1 and ADAM9 in AML development and found that NACC1 promotes the progression of AML by modulating ADAM9 expression and activating the PI3K/AKT axis.

## Materials and Methods

### Cell culture

This study used AML cell lines (HL-60, NB4, U937, KG1a, and THP1), all derived from the American Type Culture Collection (ATCC, USA). The cell lines were cultured in RPMI-1640 medium (#C11875500BT, Gibco, USA) containing 10% FBS (#900-108, Gemini, USA) and 1% penicillin-streptomycin (#C0222, Beyotime, China). The culture was kept at 5% CO2 and 37°C.

### Separation of peripheral blood mononuclear cells (PBMCs)

Peripheral blood samples from healthy donors were collected and placed in EDTA-coated tubes. A medium for mononuclear cell separation (#LDS1075, TBD, Tianjin, China) was employed to separate PBMCs and preserve them at a temperature of -80°C.

### qRT-PCR

After extracting total RNA from AML cells using TRIzol reagent (#9108, Takara, Japan), the extracted RNA was reverse transcribed into cDNA using the PrimeScript™ RT reagent kit (#RR600, Takara, Japan). The SYBR® Premix Ex Taq™ II kit (#RR820A, Takara) and the CFX Connect™ RT-qPCR system (Bio-Rad, USA) were used to conduct real-time quantitative polymerase chain reaction (RT-qPCR).

All primers were synthesized by Sangon Biotech (Shanghai, China). NACC1 primers: (F: 5′-CTCTCCCGGCTGAACTTATCAAC-3′, R: 5′-GTACACGTTGGTGCCTGTCAC-3′.). ADAM9 primers: (F: 5′-TCCATTGCTCTTAGCGACTGT-3′, R: 5′-GGGGTTCAATCCCATAACTCG-3′.). β-actin primers: (F: 5′-TGACGTGGACATCCGCAAAG-3′, R: 5′-CTGGAAGGTGGACAGCGAGG-3′).

### Western blotting

After collecting pretreated cells, they underwent three rounds of washing in phosphate-buffered saline (PBS), which was cooled beforehand. Then, total protein was extracted using ice-cold RIPA buffer (#P0013B, Beyotime, China) with PMSF (#ST505, Beyotime, China). The BCA protein concentration detection kit (#P0010, Beyotime, China) was employed to measure protein concentrations. After separating proteins via 10% SDS-PAGE gel electrophoresis, they were transferred to PVDF membranes (#IPVH00010, Millipore, USA) and then blocked with 5% skimmed milk for 2 hours. Subsequently, the membrane was covered with specific primary antibodies and placed in a refrigerator at 4 degrees for a minimum of 16 hours. The following primary antibodies were employed in this study: Cleaved PARP (#5625T, Cell Signaling Technology, USA), Cleaved-Caspase3 (#9664T, Cell Signaling Technology, USA), Cleaved-Caspase9 (#7237T, Cell Signaling Technology, USA), p-PI3K (#4228T, Cell Signaling Technology, USA), p-AKT (#4060T, Cell Signaling Technology, USA), Bcl-2 (#381702, ZENBIO, China), Bax (#R22708, ZENBIO, China), c-Myc (#343250, ZENBIO, China), p21 (#HA500156, HUABIO, China), p27kip1(#ET1608-61, HUABIO, China), AKT1/2/3 (#ET1609-51, HUABIO, China), PI3K (#ET1608-70, HUABIO, China), Cyclin D1 (#WL01435a, Wanleibio, China), Cyclin E (#WL01072, Wanleibio, China), NACC1 (#sc-376216, Santa Cruz Biotechnology, USA), ADAM9 (#ab218242, Abcam, UK), and β-actin (#BA2305, Boster, USA). Finally, a secondary antibody (Biosharp, China) was added to the membrane for an hour. The secondary antibody was incubated at a 1:4000 dilution ratio. The protein signal was evaluated using an ECL kit (EMD Millipore, USA).

### Cell infection

AML cell lines with steady NACC1 and ADAM9 knockdown were produced using recombinant lentivirus (GenePharma, Shanghai, China). Following 48 h of lentiviral infection, the fluorescence level of GFP in cells was assessed. Subsequently, AML cells were subjected to a two-week screening process using 2 µg/ml of puromycin (#P8230, Solarbio, Beijing, China), and then, the cells were harvested for subsequent analyses.

### CCK8

We used the CCK8 kit (#CA1210, Solarbio, Beijing, China) to determine cell viability. 5×10^3^ cells were put in each well of the 96-well plates. At 0/24/48/72/96 hours, 10 μL of the CCK8 reagent was added. An enzyme-labeled instrument was utilized to obtain optical density (OD) values at 450 nm. Data were sorted and analyzed. In another experiment, cells were treated with 10 μM SC79 (#HY-18749, MCE, USA) for 48 hours and 72 hours, and cell viability was measured as described above.

### Flow cytometry assay

Collected cells (1×106) were kept at 4°C overnight with 75% ethanol after three rounds of washing with cold PBS. After centrifugation the supernatant was discarded, and the cells were exposed to RNase A for 25 minutes at 37°C in darkness. Thereafter, propidium iodide (PI, Millipore) was added to the mixture. The cell cycle phase was determined utilizing FACSCaliburTM Flow cytometry (BD Biosciences) at 4°C in a dark environment.

To determine the apoptosis rate, cells (1×106) were collected and subjected to three rounds of washing and resuspending with 1 ml of PBS. Thereafter, the Annexin V APC-A and DAPI kits were used to stain cells. Finally, the CytoFLEX flow cytometer (Beckman, USA) was used to examine the stained cells. Data processing was conducted with CytExpert software (Beckman Coulter, USA).

### RNA sequencing

AML cells from control and NACC1 knockdown groups were harvested. Three times independent replication experiments were conducted in each group. Following two successive washes with sterile PBS, the total RNA for each group was extracted using TRIzol (Takara, Japan) kit. The RNA-Seq library construction and subsequent data processing were conducted by LC-Bio Technology Co. Differential expression analysis of genes was conducted between two different groups using DESeq2 software (and between two samples using edgeR). The genes with the parameter of p-value less than 0.05 and absolute fold change ≥ 2 were considered differentially expressed genes.

### Bioinformatics analysis

The expression of NACC1 (Dataset#227651_at) and ADAM9 (Dataset#1555326_a_at) in hematopoietic stem cells (HSCs) and normal AML patients were analyzed by investigating the BloodPool: AML samples and normal cells dataset of BloodSpot data. The downloaded data were imported into GraphPad Prism 8 for statistical analysis.

### Statistical analysis

Data were obtained from 3 individual experiments. Data are displayed as mean ± standard deviation (SD). GraphPad Prism 8 was used to conduct a detailed statistical evaluation of data, including but not limited to unpaired student's t-tests and one-way ANOVA. Two samples were compared using the student's t-tests, and several samples were compared using the one-way analysis of variance. Accordingly, P<0.05 was used to define statistical significance. *p <0.05, **p < 0.01, ***p <0.001, and ****p < 0.0001, and ns indicated no significant difference.

## Results

### NACC1 expression and effectiveness of knockdown in AML cells

**Figure 1:**
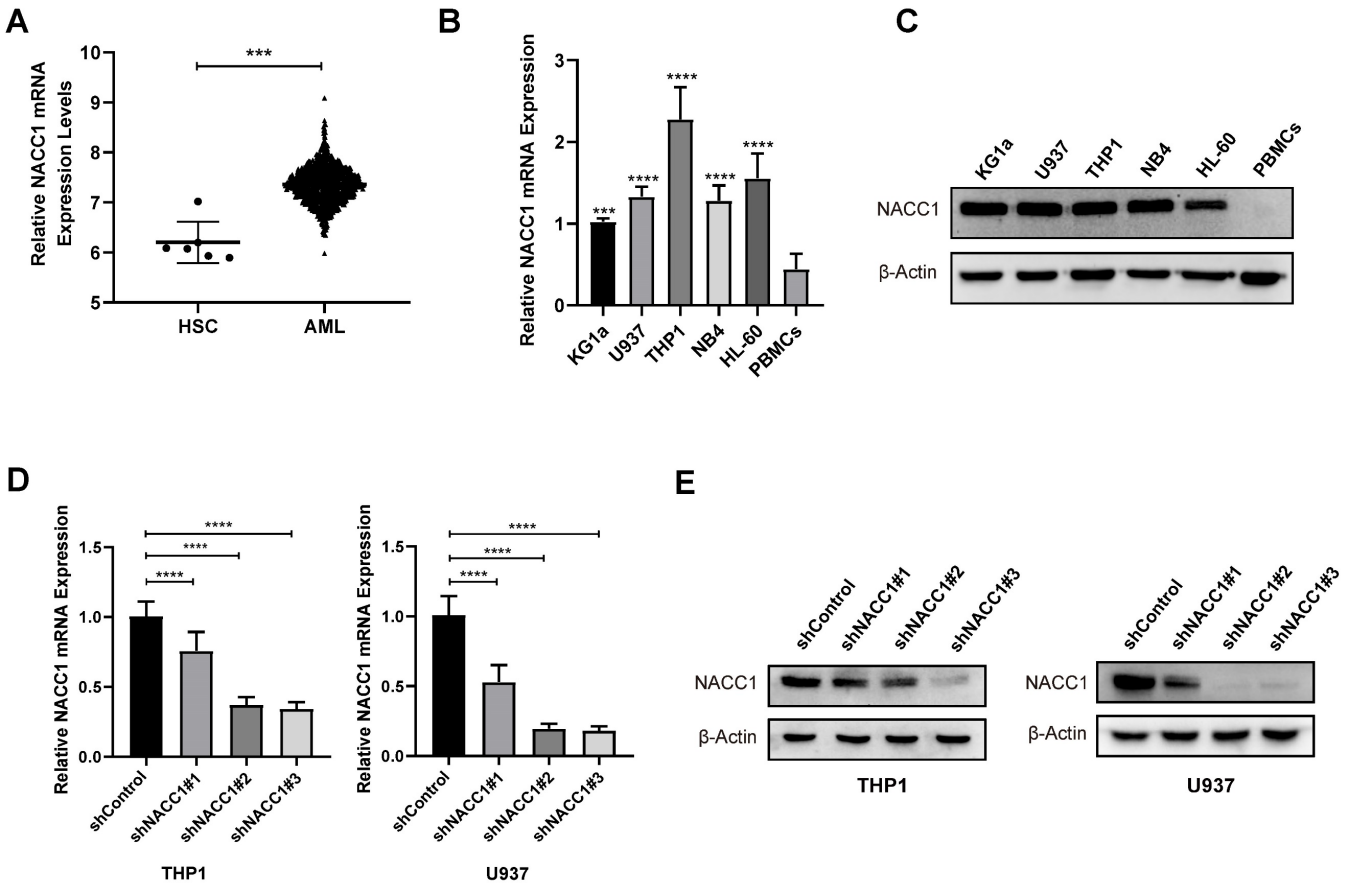
NACC1 expression and effectiveness of knockdown in AML cells. The BloodSpot database provided RNA-seq data. (A). Using qRT-PCR, the mRNA levels of NACC1 in PBMCs and AML cell lines (KG1a, U937, NB4, HL-60, and THP1) were determined (B). The protein levels of NACC1 in AML cells and PBMCs were measured using immunoblotting (C). THP1 and U937 cells were treated with different NACC1 shRNAs. The effectiveness of knockdown was evaluated using qRT-PCR (D) and immunoblotting (E).

The Bloodspot database was employed to determine the levels of NACC1 mRNA in patients with AML. We found that NACC1 expression in patients with AML is much higher than that of normal human hematopoietic stem cells (HSCs) (Fig. 1A). Subsequently, we measured NACC1 expression in various AML cell lines, including HL-60, THP1, NB4, U937, and KG1a by qRT-PCR and immunoblotting. We observed a significantly higher NACC1 expression in AML cells than in PBMCs (Fig. 1B and C). Based on these data, we selected THP1 and U937 cells with high expression of NACC1 for subsequent experimental analyses. We successfully knocked down NACC1 in THP1 and U937 cells using lentivirus and proved the effectiveness of knockdown at mRNA (Fig. 1D) and protein levels (Fig. 1E). Subsequently, we selected shRNA#3 with optimal knockdown efficacy for subsequent experiments.

### NACC1 knockdown inhibited proliferation, led to G0/G1 phase arrest, and accelerated apoptosis via the PI3K/AKT axis

**Figure 2:**
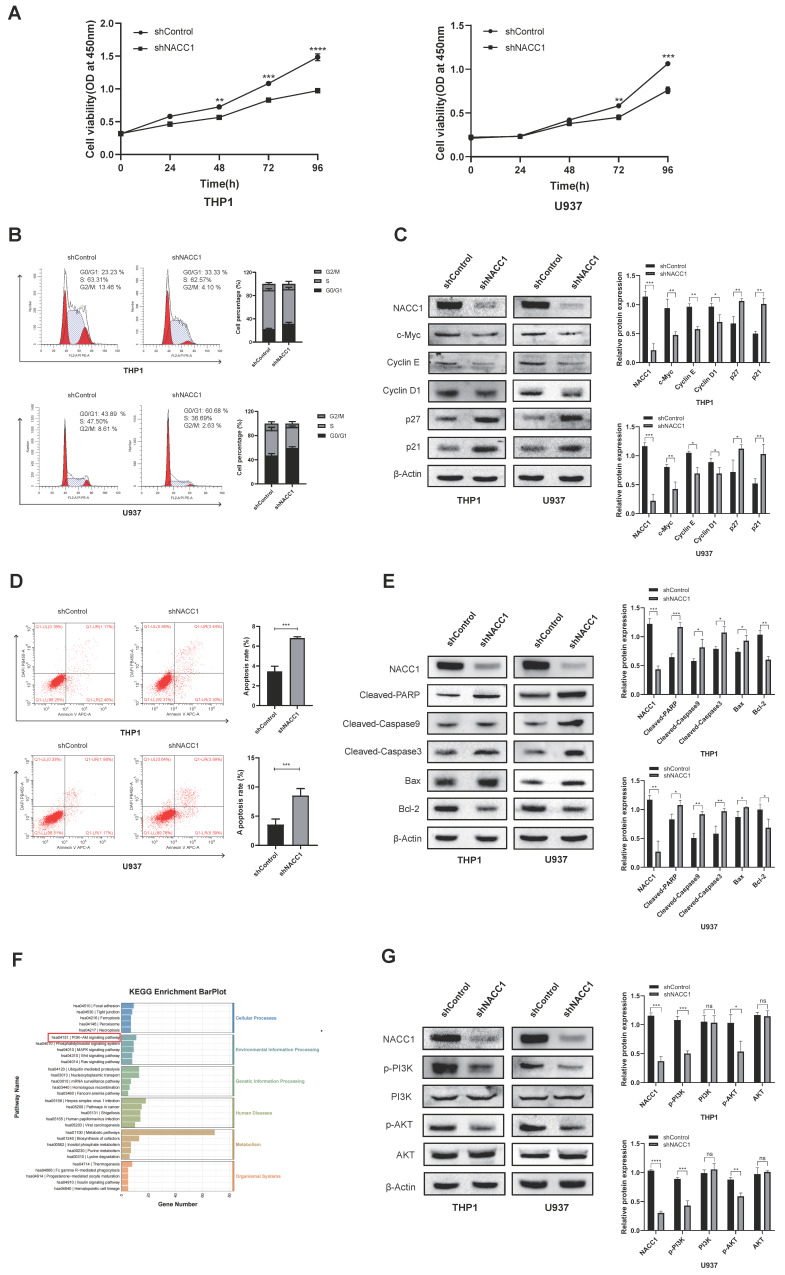
NACC1 knockdown inhibited proliferation, led to G0/G1 phase arrest, and accelerated apoptosis via the PI3K/AKT axis. CCK8 test indicated that NACC1 knockdown suppressed the proliferation of U937 and THP1 cell lines (A). Using flow cytometry, the cell cycle distribution of THP1 and U937 cells was identified (B). Cell cycle-related molecules were detected using immunoblotting (C). The apoptotic rates of THP1 and U937 cells were assessed using flow cytometry (D). Immunoblotting of proteins linked to apoptosis (E). KEGG enrichment analysis was conducted for the significant gene signatures after NACC1 knockdown in U937 cells (F). Immunoblotting was utilized to assess the levels of proteins associated with the PI3K/AKT axis (G).

To explore the exact function that NACC1 plays in AML cells, we initially employed the CCK8 assay to ascertain cell growth rates. The findings demonstrated that NACC1 knockdown markedly decelerated the proliferation of U937 and THP1 cells (Fig. 2A). We utilized flow cytometry (FCM) to assess variation in cell cycle progression. Our findings indicated that NACC1 knockdown enhanced the proportions of the G0/G1 phase in THP1 and U937 cells (Fig. 2B). Then, we measured the G0/G1 phase-related molecules and observed their changes. Immunoblotting indicated that the expression of c-Myc, Cyclin E, and Cyclin D1 reduced after NACC1 knockdown, while the levels of p21/p27 significantly elevated (Fig. 2C). Next, we used flow cytometry to investigate cells' apoptosis rate after NACC1 knockdown. Based on the results, NACC1 knockdown enhanced the apoptosis rate of AML cells (Fig. 2D). Meanwhile, Western blotting showed that the trial group with NACC1 knockdown had considerably lower expression levels of Bcl-2 compared to controls. In contrast, apoptosis-promoting molecules Bax, Cleaved-PARP, Cleaved-caspase9, and Cleaved-caspase3 all had higher expression levels in the NACC1 knockdown group (Fig. 2E). Subsequently, we conducted RNA sequencing in the NACC1 knockdown group and control group of U937 cells. Then, we conducted KEGG enrichment analysis using the sequencing data (Fig. 2F). NACC1 is abnormally expressed in cancer cells and activates the AKT-related signaling pathway, thereby exerting its biological function 44. Therefore, it is inferred that the predominant biological role of NACC1 in AML cells is achieved by activating the PI3K/AKT axis. Immunoblotting indicated that PI3K and AKT phosphorylation levels were markedly downregulated after NACC1 knockdown, which inhibited the PI3K/AKT signaling pathway (Fig. 2G). Collectively, our results indicated that the role of NACC1 in AML is modulated through the PI3K/AKT axis.

### Suppression of NACC1 expression downregulated ADAM9 expression

**Figure 3:**
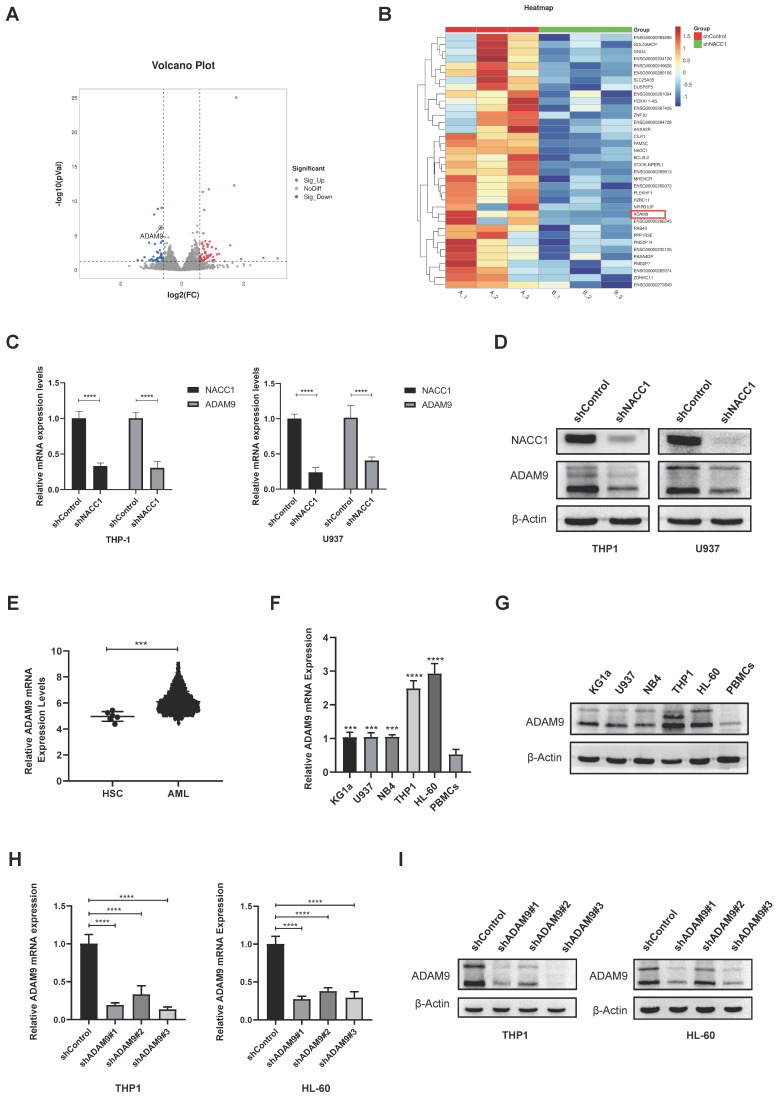
Suppression of NACC1 expression downregulated ADAM9 expression. The volcano plot depicts some of the genes expressed in the control group and NACC1 knockdown group of U937 cells. Genes with negligible changes are shown by black dots, downregulated genes are shown by blue dots, and upregulated genes are shown by red dots. |log2FC|>=1 p<0.05 (A) Heat maps revealed differential expression genes in RNA sequences between the comparison group and the NACC1 suppression group (B). qRT-PCR (C) and immunoblotting (D) were performed to investigate the effectiveness of NACC1 knockdown in THP1 and U937 cell lines and measure ADAM9 expression after NACC1 silencing. Data used in this chart are from the BloodSpot database (E). ADAM9 expression in PBMCs and 5 AML cell lines was measured by qRT-PCR (F) and immunoblotting (G). qRT-PCR (H) and immunoblotting (I) were conducted to assess the efficiency of ADAM9 knockdown in THP1 and HL-60 cells.

To unravel the downstream regulatory mechanisms underpinning the effect of NACC1 on AML cells, we conducted a detailed analysis of differentially expressed genes obtained through RNA sequencing. Sequencing analysis demonstrated that NACC1 knockdown remarkably decreased the expression levels of ADAM9 (Fig. 3A and B). Immunoblotting and qRT-PCR demonstrated that ADAM9 expression was remarkably downregulated in THP1 and U937 cell lines after NACC1 silencing (Fig. 3C and D). We hypothesized that reduced NACC1 expression substantially decreased ADAM9 expression, thereby affecting the biological function of AML. To verify this theory, we first confirmed that ADAM9 was expressed at a high level in AML cells. By analyzing the Bloodspot database, we observed that normal AML patients had considerable upregulation of ADAM9 (Fig. 3E). Our findings indicated that ADAM9 expression was higher in AML cells than in PBMCs (Fig. 3F and G). Subsequently, two cell lines THP1 and HL-60 with high ADAM9 expression were selected for the lentiviral knockdown experiment. Based on the data of qRT-PCR and immunoblotting, shRNA#3 with a better knockdown efficiency was selected for subsequent experiments (Fig. 3H and I). These results indicated that low NACC1 expression downregulated ADAM9 expression and ADAM9 was highly expressed in AML.

### The effect of ADAM9 downregulation on AML cell activity was mediated by the PI3K/AKT pathway

**Figure 4:**
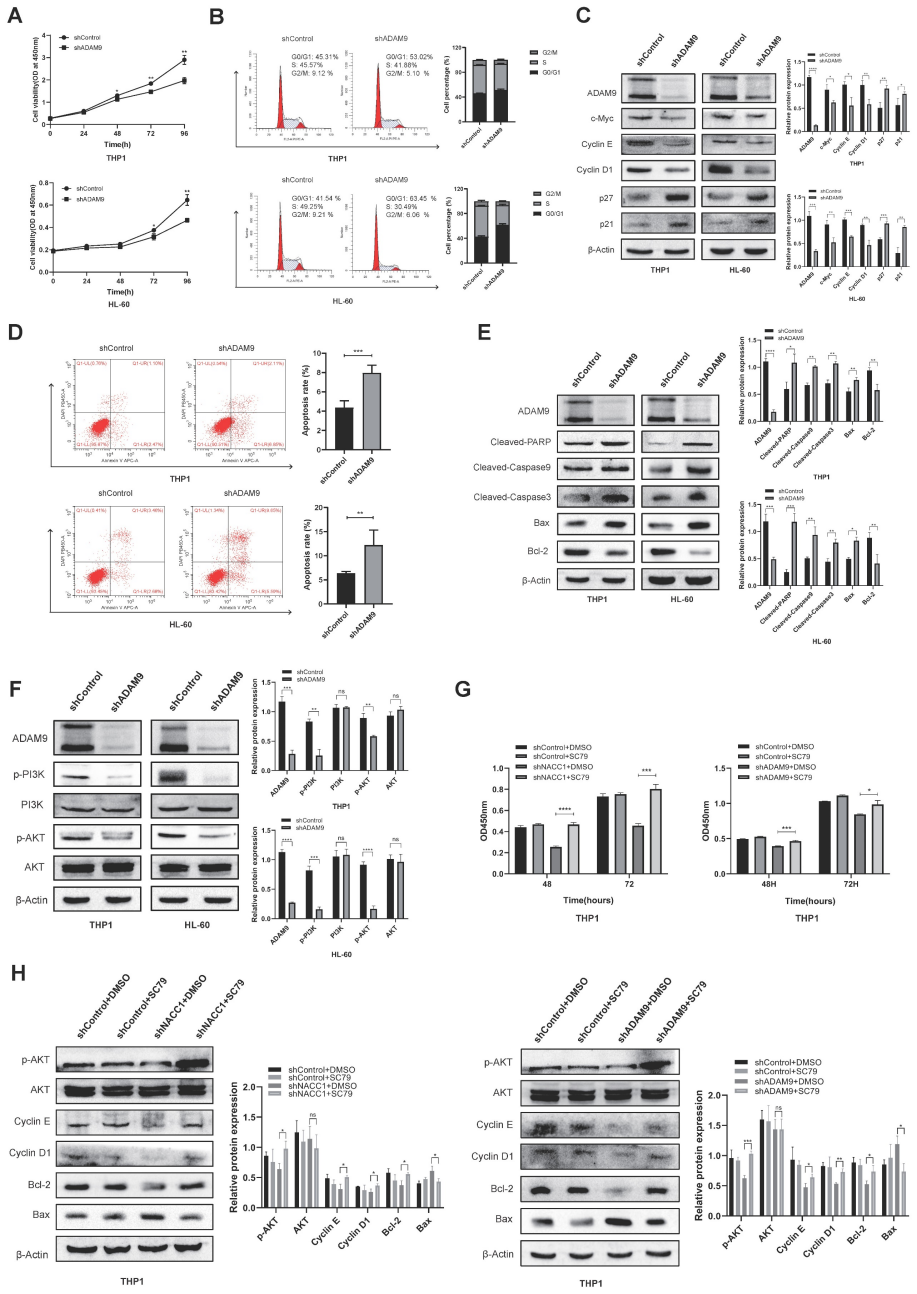
The effect of ADAM9 downregulation on AML cell activity was mediated via the PI3K/AKT axis. The growth of THP-1 and HL-60 cells was suppressed by silencing ADAM9 (A). The change in the cellular cycle was determined by FCM (B). The expression levels of proteins associated with cell cycle were assessed via WB (C). The apoptosis rates of THP1 and HL-60 cell lines were evaluated via FCM (D). The expression levels of apoptosis-related proteins were measured using immunoblotting (E). Using immunoblotting, the expression levels of proteins associated with the PI3K/AKT axis were measured (F). CCK8 assay detected the proliferation of THP1 cells treated with NACC1/ADAM9 knockdown and/or SC79 (10µM) after processing (G). Effects of AKT activator SC79 on p-Akt, cycle-related proteins, and apoptosis-related proteins (H).

Initially, we used the CCK8 assay to investigate how ADAM9 knockdown affects THP1 and HL-60 cell growth. The experimental findings indicated significant suppression of growth in both cell lines (Fig. 4A). Using flow cytometry, we discovered that ADAM9 knockdown induced the G0/G1 phase arrest (Fig. 4B). We determined the expression levels of pertinent cyclins via immunoblotting, and it was found that the protein levels of p21/p27 increased, whereas those of c-Myc, Cyclin D1 and E decreased (Fig. 4C). We examined the activity of AML cells by FCM, revealing that the apoptotic ratio of these cells was enhanced after ADAM9 silencing (Fig. 4D). Western blotting indicated that ADAM9 repression enhanced the expression of pro-apoptotic proteins, including Cleaved-PARP, Cleaved-caspase9, Cleaved-caspase3, and Bax and reduced that of Bcl-2 (Fig. 4E). Then, we explored the mechanism of ADAM9 in AML and investigated the effect of ADAM9 on the PI3K/AKT axis by knocking down ADAM9. The results showed that PI3K phosphorylation and AKT phosphorylation were downregulated, indicating that ADAM9 knockdown suppressed the activity of the PI3K/AKT axis (Fig. 4F). We treated NACC1 knockdown THP1 cells and ADAM9 knockdown THP1 cells with the AKT activator SC79 to determine whether the carcinogenic effects of NACC1 and ADAM9 are mediated by the PI3K/AKT pathway. The findings demonstrated that the addition of SC79 markedly enhanced the proliferation of cells with NACC1 and ADAM9 knockdown (Fig. 4G). In the knockdown cell lines, SC79 enhanced the expression level of p-AKT protein and altered the expression of apoptosis-related proteins Bcl-2 and Bax, as well as Cyclin E and Cyclin D1 (Fig. 4H). In conclusion, ADAM9 knockdown can promote AML cell apoptosis, decelerate AML cell growth, and change cell cycle distribution via the PI3K/AKT pathway.

## Discussion

The etiology of AML remains widely unknown, and the prevalence of AML has been rising in recent years 45, 46. AML mostly affects older people, with a median diagnosis age of almost 70 years 47. While optimizing drug dosage and combinations of new drugs during induction therapy can prolong the survival of patients with newly diagnosed AML 48-50, this strategy is less effective in older patients, and the recurrence rate remains high 51. The precise etiology of AML remains elusive in the majority of patients, and to date, there is insufficient compelling evidence to endorse any single method as the gold standard for care 52, 53. Accordingly, it is of great importance to delve into promising therapeutic targets. Our study clarified the mechanism by which NACC1 and ADAM9 are involved in the pathogenesis of AML. The experiment employed THP1 and U937 cells from AML-M5, with HL-60 cells from AML-M2. NACC1 and ADAM9 are markedly aberrant expressed in these three cell lines, which are often used in AML research. Meanwhile, our results corroborate the pivotal role of the NACC1/ADAM9/PI3K/AKT axis in the proliferation, apoptosis, and cell cycle of AML cells. Therefore, it provides a new idea for finding new therapeutic and diagnostic targets for AML.

NACC1 is overexpressed in various malignancies, correlating positively with tumor progression and recurrence, suggesting a poor prognosis 54. Gao *et al.* compared differential genes in ovarian cancer cells after NACC1 overexpression and knockdown. They found that NACC1 was strongly associated with multiple genes and pathways associated with cancer, indicating that it is crucial for the growth of tumors 55. Furthermore, high expression of NACC1 has been correlated with low overall survival rates of patients suffering from cervical cancer 56. Our study confirmed that NACC1 is overexpressed in AML cell lines. NACC1 knockdown suppressed the proliferation ability of AML cells, increased the apoptosis rate, and led to cell cycle arrest. Previous reports have elucidated a close association between the abnormal expression of NACC1 in tumor cells and ATK-related signaling pathways 44, 57. Accumulating data suggest that the PI3K/AKT/mTOR axis is critical for the development and progression of AML 58, 59. Our KEGG enrichment analysis results indicated that NACC1 is closely associated with the PI3K/AKT signaling pathway. Our results suggested that NACC1 knockdown suppressed the PI3K/AKT pathway. Signaling pathways such as MAPK, Wnt, and Ras, which were enriched by KEGG results, regulate various biological effects, such as cell proliferation and differentiation, and play an important role in AML. Many enzymes involved in these pathways are considered to be potential targets for treating cancer 60, 61. Thus, these results provide invaluable clues for further exploration of the mechanism of NACC1 in future experiments on AML. Briefly, our results indicated that NACC1 promotes the development of AML. Thus, NACC1 is predicted to be a useful target for the treatment of AML. It may be useful as a tumor marker to assess the prognosis of AML patients.

According to reports, the ADAMs protein family constitutes a promising family of therapeutic targets in cancer 62. Previous studies have identified that ADAMs family members are expressed in various hematological malignancies 63. ADAM9 is overexpressed in several tumors and affects tumor progression through different mechanisms 64. Several studies have confirmed that abnormal ADAM9 expression can efficiently stimulate the growth and invasion of malignant cells 65, 66. Furthermore, ADAM9 is a transcription factor that encourages angiogenesis in esophageal cancer 67. The expression and functional role of ADAM9 in AML remain unclarified. In our study, we discovered that ADAM9 is abnormally expressed in AML cells and affects cell survival by triggering the PI3K/AKT axis. Meanwhile, we found that NACC1 knockdown in AML cells downregulates ADAM9 expression. Although the precise mechanism is yet to be fully understood, these results demonstrate the importance of ADAM9 in the treatment of AML.

Taken together, our findings demonstrated that NACC1 and ADAM9 contribute to the development of AML. Nonetheless, there are some shortcomings to this study. The lack of clinical specimens did not allow the assessment of NACC1 and ADAM9 expression in primary isolates. Future endeavors can help overcome these limitations and investigate the specific regulatory role of NACC1 in ADAM9.

## Conclusion

In conclusion, this study revealed that NACC1 and ADAM9 were abnormally overexpressed in AML cell lines. The abnormal expression of NACC1 modulated the expression of ADAM9, subsequently activating the PI3K/AKT axis, enhancing the proliferation of AML cells, accelerating cell cycle progression, and inhibiting apoptosis.

## Abbreviations

AML: acute myeloid leukemia
NACC1: Nucleus accumbens-associated protein 1
ADAM9: A disintegrin and metalloproteinase 9
BTB Domain: Broad Complex, Tramtrack and Bric a Brac Domain
qRT-PCR: quantitative real-time PCR
PBS: Phosphate Buffered Saline
CCK8: Cell counting kit-8
OD: optical density
FCM: Flow cytometry
FBS: fetal bovine serum
P27: p27kip1
PBMCs: peripheral blood mononuclear cells
p-PI3K: phosphorylated PI3K
p-AKT: phosphorylated AKT
